# Regulatory domain or CpG site variation in *SLC12A5*, encoding the chloride transporter KCC2, in human autism and schizophrenia

**DOI:** 10.3389/fncel.2015.00386

**Published:** 2015-10-12

**Authors:** Nancy D. Merner, Madison R. Chandler, Cynthia Bourassa, Bo Liang, Arjun R. Khanna, Patrick Dion, Guy A. Rouleau, Kristopher T. Kahle

**Affiliations:** ^1^Harrison School of Pharmacy, Department of Drug Discovery and Development, Auburn UniversityAuburn, AL, USA; ^2^Department of Neurology and Neurosurgery, Montreal Neurological Hospital and Institute, McGill UniversityMontréal, QC, Canada; ^3^Department of Biological Chemistry and Molecular Pharmacology (BCMP), Harvard Medical SchoolBoston, MA, USA; ^4^Department of Neurosurgery, Boston Children’s Hospital and Harvard Medical SchoolBoston, MA, USA; ^5^Manton Center for Orphan Disease Research, Boston Children’s HospitalBoston, MA, USA

**Keywords:** KCC2, NKCC1, GABA, neurodevelopmental disorders, autism, schizophrenia

## Abstract

Many encoded gene products responsible for neurodevelopmental disorders (NDs) like autism spectrum disorders (ASD), schizophrenia (SCZ), intellectual disability (ID), and idiopathic generalized epilepsy (IGE) converge on networks controlling synaptic function. An increase in KCC2 (*SLC12A5*) Cl^−^ transporter activity drives the developmental GABA excitatory-inhibitory sequence, but the role of KCC2 in human NDs is essentially unknown. Here, we report two rare, non-synonymous (NS), functionally-impairing variants in the KCC2 C-terminal regulatory domain (CTRD) in human ASD (R952H and R1049C) and SCZ (R952H) previously linked with IGE and familial febrile seizures, and another novel NS KCC2 variant in ASD (R1048W) with highly-predicted pathogenicity. Exome data from 2517 simplex families in the ASD Simon Simplex Collection (SSC) revealed significantly more KCC2 CTRD variants in ASD cases than controls, and interestingly, these were more often synonymous and predicted to disrupt or introduce a CpG site. Furthermore, full gene analysis showed ASD cases are more likely to contain rare KCC2 variants affecting CpG sites than controls. These data suggest genetically-encoded dysregulation of KCC2-dependent GABA signaling may contribute to multiple human NDs.

## Introduction

Neurodevelopmental disorders (NDs) encompass a wide range of diseases, all of which feature some element of impaired brain development, and are associated with cognitive, neurological, and/or psychiatric dysfunction (Rubenstein, 2011). Common NDs include intellectual disability (ID), autism spectrum disorder (ASD), schizophrenia (SCZ), and epilepsy (Guilmatre et al., 2009; Bozzi et al., 2012), and although classified into distinct disease categories, these disorders show phenotypic overlap and shared genetic risk factors (Mitchell, 2011; Coe et al., 2012). The genetic architecture of NDs is complex, with oliogenic contributions converging on the disruption of the structure, function, and/or plasticity of neuronal networks (Pescosolido et al., 2012). Genomics has shed insight into mechanisms underlying the overlap among the NDs, with copy number variant, exome sequencing, and genome-wide association study data suggesting a spectrum of neurodevelopmental pathology indexed by mutational load or severity (Krystal and State, 2014). Emerging evidence has shown many of the encoded gene products responsible for individual NDs converge on a relatively limited number of protein-protein interaction networks that operate in the same molecular processes, such as those controlling synaptic structure and function (Gilman et al., 2011; Voineagu et al., 2011; O’Roak et al., 2012; EPGP Collaborative et al., 2013; de Rubeis et al., 2014; Krumm et al., 2014).

Small, diverse populations of inhibitory GABAergic interneurons regulate the activity of excitatory neurons and their involved circuits. If inhibition is impaired, disturbance in excitatory/inhibitory balance can lead to the dysfunction of cognitive processes (Braat and Kooy, 2015). A common feature among different NDs is impaired GABAergic inhibition (Hashimoto et al., 2008; Kang and Macdonald, 2009; Schmidt and Mirnics, 2014), and neuronal hyperexcitability has been implicated in the pathogenesis of ASD (Coghlan et al., 2012), SCZ (Lewis et al., 2012), Rett syndrome (Medrihan et al., 2008; Chao et al., 2010), Tourette syndrome (Kalanithi et al., 2005), tuberous sclerosis (Talos et al., 2012), and neurofibromatosis type I (Cui et al., 2008). The strong bidirectional association between NDs and epilepsy suggests impaired GABAergic inhibition as a potential pathogenic mechanism of mutual susceptibility (Tuchman and Rapin, 2002; Brooks-Kayal, 2010; Chang et al., 2011; Deidda et al., 2014).

Ionotropic GABA_A_Rs are ligand-gated Cl^−^ channels, and the post-synaptic response to GABA_A_R activation is significantly modulated by the intraneuronal concentration of Cl^–^ ([Cl^–^]_i_). In immature neurons, [Cl^−^]_i_ is sufficiently high that GABA_A_R activation triggers giant depolarizing potentials, which characterize early network activity and stimulate Ca^2+^-dependent synaptogenesis (Ben-Ari et al., 1989; Belhage et al., 1998). A post-natal increase in the functional expression of KCC2 (*SLC12A5*), a cation-Cl^−^ cotransporter (CCC) mediating Cl- efflux, lowers [Cl^−^]_i_ in post-synaptic neurons such that GABA_A_R activation elicits membrane hyperpolarization and fast synaptic inhibition (Kaila et al., 2014). KCC2 is required for the normal developmental GABA excitatory-inhibitory sequence, and KCC2 deficiency disrupts normal brain development and results in network hyperexcitability (Hubner et al., 2001; Hekmat-Scafe et al., 2006, 2010; Tanis et al., 2009; Bellemer et al., 2011).

Abnormal functional expression of KCC2 or its Cl^−^ importing cousin NKCC1, and associated impairment of GABA inhibition, have been documented in rodent models of multiple different NDs [see Table 1 in Deidda et al. (2014)]. For example, in mouse models of Fragile X syndrome, the most common genetic cause of human autism, there is a significantly delayed developmental switch to GABAergic inhibition due to prolonged elevation in neuronal [Cl^−^]_i_ due in part to decreased KCC2 expression (Lemonnier et al., 2013), and blockade of up-regulated NKCC1-mediated Cl^−^ import in this context normalizes [Cl^−^]_i_, electrophysiological responses, and autistic-like behaviors in these mice (Tyzio et al., 2014), and improves autistic behaviors in humans (Lemonnier et al., 2012). However, the role of KCC2 in the human nervous system and in NDs is essentially unknown. Identification of such variants could elucidate the molecular pathophysiology of these diseases and identify therapeutic targets.

**Figure 1:**
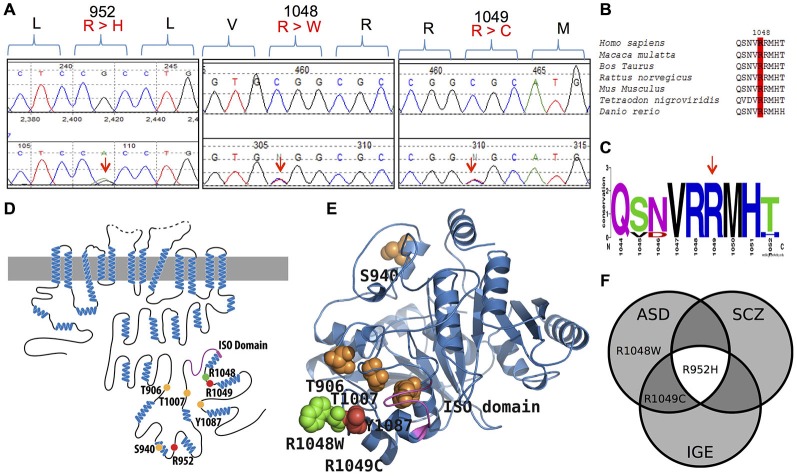
***KCC2* (*SLC12A5*) variants in human autism spectrum disorder (ASD) and schizophrenia (SCZ). (A)** DNA chromatograms illustrating the detection of KCC2 variants in ASD (c.2855 G > A [p.R952H]; c.3145 C > T [p.R1049C]; and c.3142 C > T [p.R1048W]); and SCZ (p.R952H) via Sanger sequencing. **(B,C)** Evolutionary conservation of amino acid p.R1048; and conservation of amino acids p.R952 and p.R1049 shown in Kahle et al. (2014). **(D)** Schematic representation of KCC2 (human). Orange dots indicate the positions of the known critical phospho-regulatory residues p.T906, p.S940, p.T1007, and p.Y1087 (reviewed in Chamma et al., 2012; Kahle et al., 2013); Pink region denotes the KCC2 “ISO” domain, required for hyperpolarizing GABAergic transmission (Acton et al., 2012). Red dots depict the identified IGE mutations, p.R952 and p.R1049; green dots depict the identified ASD variants, p.R952H, p.R1049C, and p.R1048W; yellow dots indicate the identified SCZ variant p.R952H. **(E)** The modeled structure of the human KCC2 C-terminal domain (CTRD), based on homology modeling by I-TASSER (Roy et al., 2010) using a prokaryotic member of the CCC family (PDB code 3g40) (for details, see “Materials and Methods” Section). Color scheme same as in **(D)**. Note the proximity of the novel p.R1048W ASD variant and the previously described KCC2 IGE variants, as well as their relation to important regulatory residues and domains. **(F)** Venn diagram showing overlap of KCC2 variants in multiple neurodevelopmental phenotypes (IGE, ASD, and SCZ) that exhibit dysfunctional GABA signaling (Deidda et al., 2014).

The KCC2 C-terminal regulatory domain (CTRD) is a critical region of transporter function, and contains multiple phosphorylated residues (such as Thr906/Thr1007 and Ser940 (reviewed in Blaesse et al., 2009; Chamma et al., 2012; Kahle et al., 2013; Medina et al., 2014); and functional domains (e.g., Acton et al., 2012) that establish context-appropriate transport activity (de Los Heros et al., 2014; see Figure 1). We recently identified an enrichment of KCC2 non-synonymous (NS) alleles in French Canadian (FC) idiopathic generalized epilepsy (IGE) cases compared to controls that included two rare and functional IGE risk alleles (KCC2 R952H and R1049C; Kahle et al., 2014). Both of these variants decrease KCC2-mediated Cl^−^ extrusion and render neuronal GABA activity more depolarized by decreasing the amount of Ser940 stimulatory phosphorylation (Kahle et al., 2014). KCC2 R952H was also identified as a cause of an inherited form of febrile seizures in an Australian family, and shown to impair dendritic spine formation (Puskarjov et al., 2014).

Here, we sought to determine if risk alleles in the KCC2 CTRD (amino acids 894–1086; NP_065759) were present in large FC cohorts of ASD, SCZ, or ID by utilizing a similar targeted genetic sequencing approach we successfully utilized for IGE (Kahle et al., 2014). We speculated that genetic mutations and/or functionally impairing variants in the KCC2 CTRD might contribute to NDs.

## Materials and Methods

### Clinical Sampling

The three disease cohorts of ASD, SCZ, and ID, were established as a part of the Synapse to Disease (S2D) project initiated by Dr. Guy Rouleau at Montreal Neurological Institute and McGill University in order to identify genes that cause or predispose to numerous disorders of brain development. The sample characteristics of these FC cohorts have previously been described (Gauthier et al., 2005; Hamdan et al., 2009; Awadalla et al., 2010).

### KCC2 C-Terminus Targeted Screening

We implemented a targeted DNA Sanger sequencing approach to screen the 3′ end of *SLC12A5* that encodes the CTRD of KCC2 (Kahle et al., 2014). Specifically, we targeted the coding nucleotides in exons 21–25 of *SLC12A5* [NM_020708.4 (NP_065759 amino acids 894–1086) or NM_001134771 (NP_001128243 amino acids 917–1110)] by following the same protocol outlined in Kahle et al. (2014); a total of 427 ASD, 143 SCZ and 190 ID cases were screened. A total of 1214 matched controls were previously screened for mutations in the targeted region of KCC2 (Kahle et al., 2014).

### Protein Sequence Alignment and *In Silico* Prediction Programs

ClustalW (Larkin et al., 2007) and WebLogo (Crooks et al., 2004) were used to align different orthologues of the KCC2 to determine the evolutionary conservation of the novel KCC2 variant, R1048W (Figures 1B,C). Conservation of KCC2 R952H and R1049C were previously demonstrated (Kahle et al., 2014). Conservation of the KCC2 protein was determined by aligning the following orthologues: *Homo sapiens* (NP_065759), *Macaca mulatta* (XM_001104494.2_prot), *Bos Taurus* (NP_001193309), *Rattus norvegicus* (NP_599190), *Mus musculus* (NP_065066), *Tetraodon nigroviridis* (ENSTNIT00000021299), and *Danio rerio* (ENSDART00000009569). Regarding the WebLogo output, the y-axis serves as a means of determining relative conservation and is not an actual measurement; the height of each stack at each amino acid position is relative to the overall conservation at that position, the height of the letters within each stack indicate the relative frequencies for each amino acid possibility, and the width of each stack corresponds to the proportion of valid readings at that position (indicating if sequence gaps exist between the shown amino acids). The effects of amino acid substitutions on protein function were predicted using MutationTaster (Schwarz et al., 2010), Panther (Mi et al., 2005), and Polyphen-2 (Adzhubei et al., 2010).

### Exome Sequencing Analysis

Iossifov et al. (2014) recently reported the whole exome sequencing of 2517 ASD simplex families from the Simon Simplex Collection (SSC; Fischbach and Lord, 2010). They carried out a *de novo* mutation analysis that generated an extensive list of *de novo* variants and recurrently hit genes that could be subdivided with different clinical phenotypes (Iossifov et al., 2014). These exome sequencing data are now available through the NDAR (National Database of Autism Research); we were granted access to variant calling files to further assess *SLC12A5*.

Our analysis involved filtering for sample type to include only probands (.p1), accession number (NM_001134771) to include only *SLC12A5*, and “_par-races_” (we either selected “white, white” or “african-amer, african-amer”) to include probands with either two Caucasian parents (EA ASD cases) or two African American parents (AA ASD cases), respectively. According to Supplementary Table 1 in Iossifov et al. (2014), there were 1892 EA ASD cases and 82 AA ASD cases. We carried out a rare variant (<1% MAF) analysis; therefore, we filtered out SNPs (single nucleotide polymorphisms) with a MAF >1% in each ethnic group. This resulted in a list of rare coding variants for the full gene and our targeted gene region. Furthermore, we used the EA and AA Exome Variant Server (EVS) data (NHLBI GO Exome Sequencing Project)^1^ as our ethnic controls; there were a total of 4300 EA controls and 2203 AA controls exome sequenced. We generated lists of rare variants that were detected in each control cohort.

**Table 1:** *SLC12A5* **variants detected in the FC ASD cohort through the targeted screening of the C-terminus**.

Variants detected in the screened region			Detection of variants in Quebec ASD cohort			FC population controls (Total: 1214 controls)			
NM_020708.4 NP_065759	NM_001134771.1 NP_001128243	rs ID	Number of probands	Number of alleles	Allele frequency (%)	Number of alleles	Allele frequency (%)	*p*-value	Odds ratio
c.2855 G > A	c.2924 G > A	rs142740233	2/427	2/854	0.23	5/2428	0.21	1.00	1.14
p.R952H	p.R975H								CI_95_[0.1–7.0]
c.2961 G > A	c.3030 G > A	rs550491448	1/427	1/854	0.12	0/2428	0.00	0.26	Inf
p.P987P	p.P1010P								CI_95_[0.1-Inf]
c.3142 C > T	c.3211C > T	rs369042030	1/427	1/854	0.12	0/2428	0.00	0.26	Inf
p.R1048W	p.R1071W								CI_95_[0.1-Inf]
c.3145 C > T	c.3214 C > T	–	1/427	1/854	0.12	1/2428	4.12 × 10^−4^	0.45	2.84
p.R1049C	p.R1072C								CI_95_[0.0–223.0]
Total number of variants detected			5	5	–	6	–	0.17	2.37
									CI_95_[0.6–9.4]

### Statistical Analysis

All statistical genetic analysis was carried out using the program R (version 2.15.1). Fisher tests or Mantel-Haenzel Chi Squared tests were carried out to generate the *p*-values and odds ratios where appropriate.

## Results

Using Sanger sequencing, we examined the 3′ end of *SLC12A5* that encodes the KCC2 CTRD (amino acids 894–1086; NP_065759) in three large FC disease cohorts of ASD, SCZ, or ID that were collected as part of the S2D project (see “Materials and Methods” Section). In contrast to our previous analysis in IGE which identified an enrichment of KCC2 NS CTRD alleles in IGE cases compared to controls (*p-value* = 7.50 × 10^−3^; Kahle et al., 2014), analysis of our initial ASD, SCZ or ID sequencing results did not show an enrichment of NS KCC2 CTRD alleles in cases (Table 1). Interestingly, however, three different heterozygous and NS KCC2 variants were detected in the ASD cohort; these included the two previously-identified IGE risk variants, R952H and R1049C (Kahle et al., 2014), and R1048W (Figure 1 and Table 1).

R1048 in KCC2 is a highly conserved residue (Figure 1), and a substitution for a tryptophan at this position is predicted to be highly pathogenic using multiple *in silico* bioinformatics programs (Table 2). This variant is extremely rare; it was not detected in 2428 FC alleles, but this number of controls was too small to generate a significant *p*-value (Table 1). Indeed, power analysis determined that 50,000 control alleles would need to be genotyped, assuming the allele frequencies remain the same and a *p*-value of 0.0167 is significant (after a Bonferroni correction). This limitation is common when identifying rare genetic risk factors, and also relevant for the R1049C variant (Table 1), where 100,000 control alleles would need to be genotyped to reach significance. R952H was previously determined to have an allele frequency of 0.66% in the FC IGE cohort (Kahle et al., 2014) compared to 0.21% in FC controls and 0.23% in the FC ASD cohort (Table 1). One SCZ patient was also determined to carry R925H, corresponding to an allele frequency of 0.35% (Table 3). Two heterozygous synonymous variants were also detected in our screening, including P987P in an ASD patient (Table 1), and D935D in an SCZ patient (Table 3).

**Table 2:** **Predicted pathogenicity of the novel KCC2 (*SLC12A5*) variant**.

Variant name		Prediction programs			
NM_020708.4 NP_065759	NM_001134771.1 NP_001128243	Mutation taster		Panther	Polyphen
c.3142 C > T	c.3211C > T	Disease	P (probability):	P_deleterious_ = 0.80942	Possibly damaging
p.R1048W	p.R1071W	causing*	0.9999		(0.813)

*Predicted specifically to disrupt the function of the last cytoplasmic domain.

**Table 3:** *SLC12A5*** variants detected in the SCZ cohort through the targeted screening of the C-terminus**.

Variants detected in the screened region			Detection of variants in SCZ cohort			FC population controls (Total: 1214 controls)			
NM_020708.4 NP_065759	NM_001134771.1 NP_001128243	rs ID	Number of probands	Number of alleles	Allele frequency (%)	Number of alleles	Allele frequency (%)	*p*-value	Odds ratio
c.2805 T > C	c.2874 T > C	rs151293924	1/143	1/286	0.35	0/2428	0.00	0.11	Inf
p.D935D	p.D958D								CI_95_[0.2-Inf]
c.2855 G > A	c.2924 G > A	rs142740233	1/143	1/286	0.35	5/2428	0.21	0.50	1.70
p.R952H	p.R975H								CI_95_[0.0–15.3]
Total number of variants detected			2	2	–	6*	–	0.20	2.85
									CI_95_[0.3–16.1]

*A total of 6 variants were detected in FC control cohort [5 R975H and 1 R1072C (which is not shown in this table)].

From a functional standpoint, both R952H and R1049C impair KCC2 transporter activity (Kahle et al., 2014); they significantly decrease KCC2-mediated Cl^−^ extrusion capacity in neurons, render EGly less hyperpolarized compared to WT KCC2, decrease the level of stimulatory phosphorylation of Ser940, act in a dominant-negative manner consistent with the known oligomerization of KCC2 molecules, and decrease transporter plasmalemmal expression (R952H) or lower the intrinsic activity of transporters at the cell surface (R1049C) (Kahle et al., 2014). We anticipate these variants function similarly in ASD; however, the phenotypic outcomes of any effect of these variants are likely dependent on the combination of other risk alleles within each individual patient. Considering the proximity of R1048 to R1049, and the fact that both variants substitute an arginine, we assume the functional effects of R1048W on KCC2 would be similar to that of R1049C (Kahle et al., 2014).

Overall, the data generated from the targeted screening of 3′ end of *SLC12A5* in FC cases and controls did not reach statistical significance, possibly due to the rare nature of the variants and the size of the FC cohorts (Table 4). However, when the SSC and EVS exome sequencing data was considered, the combined analysis indicated there was an enrichment of all coding KCC2 CTRD variants in ASD cases compared to controls (*p-value* = 0.03; Table 4). In fact, when subdividing the variants into various groups, we determined that ASD cases actually had significantly more synonymous variants compared to controls (*p-value* = 0.02), as well as variants that either disrupted or introduced a CpG site (*p-value* = 6.8 × 10^−3^; Table 4). Upon full gene analysis (using solely exome sequencing data), ASD cases were determined to have a higher percentage of rare *SLC12A5* variants that affect a CpG site compared to controls (Tables 5, 6), suggesting a possible epigenetic effect on gene expression through variation in methylation patterns.

**Table 4:** ***SLC12A5* variants detected in the FC and SSC EA and AA cohorts in the targeted region of the C-terminus**.

	Cohort						Combined	
Variant type	Ethnicity	Group	Size	Number of rare variants transported	*p* value	Odds ratio	*p* value	Odds ratio
All	FC	ASD Cases	427	5	0.17	2.37 CI_95_[0.6–9.4]	0.03	2.00 CI_95_[1.1–3.6]
		Controls	1214	6				
	EA	ASD Cases	1892	12	0.09	1.95 CI_95_[0.8–4.5]
		EVS Controls	4300	14
	AA	ASD Cases	82	2	0.35	1.67 CI_95_[0.2–6.8]
		EVS Controls	2203	32
Non-synonymous	FC	ASD Cases	427	4	0.30	1.90 CI_95_[0.4–8.0]	0.31	1.53 CI_95_[0.8–3.1]
		Controls	1214	6
	EA	ASD Cases	1892	9	0.37	1.46 CI_95_[0.6–3.6]
		EVS Controls	4300	14
	AA	ASD Cases	82	0	1.00	0.00 CI_95_[0.0–29.7]
		EVS Controls	2203	5
Synonymous	FC	ASD Cases	427	1	0.26	Inf CI_95_[0.1-Inf]	0.02	4.93 CI_95_[1.7–14.8]
		Controls	1214	0
	EA	ASD Cases	1892	3	0.02	Inf CI_95_[0.9-Inf]
		EVS Controls	4300	0
	AA	ASD Cases	82	2	0.28	2.00 CI_95_[0.2–8.1]
		EVS Controls	2203	27
CpG site disrupted or gained	FC	ASD Cases	427	5	0.17	2.37 CI_95_[0.6–9.4]	6.8 × 10^−3^	2.5 CI_95_[1.3–4.7]
		Controls	1214	6
	EA	ASD Cases	1892	11	0.02	2.78 CI_95_[1.0–7.6]
		EVS Controls	4300	9
	AA	ASD Cases	82	2	0.31	1.85 CI_95_[0.2–7.5]
		EVS Controls	2203	29
CpG site not disrupted or gained	FC	ASD Cases	427	0	1.00	0.00 CI_95_[0.0-Inf]	0.71	0.42 CI_95_[0.0–3.6]
		Controls	1214	0
	EA	ASD Cases	1892	1	0.67	0.45 CI_95_[0.0–4.1]
		EVS Controls	4300	5
	AA	ASD Cases	82	0	1.00	0.00 CI_95_[0.0–65.6]
		EVS Controls	2203	3

**Table 5:** **Full gene rare variant (MAF < 1%) analysis of *SLC12A5* using the SSC exome sequencing data**.

	Cohort						Combined	
Variant type	Ethnicity	Group	Size	Number of rare variants transported	*p* value	Odds ratio	*p* value	Odds ratio
All	EA	ASD Cases	1892	50	1.00	1.00 CI_95_[0.7–1.4]	0.76	1.06 CI_95_[0.8–1.4]
		EVS Controls	4300	114
	AA	ASD Cases	82	9	0.30	1.44 CI_95_[0.6–2.9]
		EVS Controls	2203	168
Non-synonymous	EA	ASD Cases	1892	21	0.68	1.11 CI_95_[0.6–1.9]	0.98	1.04 CI_95_[0.6–1.8]
		EVS Controls	4300	43
	AA	ASD Cases	82	0	1.00	0.00 CI_95_[0.0–4.8]
		EVS Controls	2203	23
Synonymous	EA	ASD Cases	1892	29	0.73	1.08 CI_95_[0.7–1.7]	0.38	1.20 CI_95_[0.8–1.8]
		EVS Controls	4300	61
	AA	ASD Cases	82	9	0.18	1.67 CI_95_[0.7–3.4]
		EVS Controls	2203	145
CpG site disrupted or gained	EA	ASD Cases	1892	41	0.22	1.29 CI_95_[0.9–1.9]	0.09	1.37 CI_95_[1.0–1.9]
		EVS Controls	4300	72
	AA	ASD Cases	82	8	0.15	1.76 CI_95_[0.7–3.7]
		EVS Controls	2203	122
CpG site not disrupted or gained	EA	ASD Cases	1892	9	0.05	0.5 CI_95_[0.2–1.0]	0.06	0.50 CI_95_[0.3–1.0]
		EVS Controls	4300	42
	AA	ASD Cases	82	1	1.00	0.58 CI_95_[0.0–3.5]
		EVS Controls	2203	46

**Table 6:** **ASD case/control comparisons of the total number of variants that affect a CpG site vs. do not affect a CpG site from the full gene analysis**.

Cohort		Total number of rare variants in *SLC12A5*	Variants that disrupt or gain a CpG site		Variants that do not disrupt or gain a CpG site				Combined	
			Number	Percentage (%)	Number	Percentage (%)	*p* value	Odds ratio	*p* value	Odds ratio
EA	ASD Cases	50	41	82	9	18	0.02	2.64 CI_95_[1.1–6.8]	0.01	2.71 CI_95_[1.3–5.8]
	EVS Controls	114	72	63	42	37
AA	ASD Cases	9	8	89	1	11	0.45	3.00 CI_95_[0.4–136.6]
	EVS Controls	168	122	73	46	38

## Discussion

The identification of KCC2 NS genetic variants in ASD and SCZ cases that involve evolutionary conserved residues, are predicted to be pathogenic, and have either previously been shown [KCC2 R952H and R1049C (Kahle et al., 2014; Puskarjov et al., 2014)] or anticipated (KCC2 R1048W) to impact transporter function, trafficking, and/or regulatory phosphorylation, suggest that genetically-encoded impairment of KCC2 function may be risk factors for, or contribute to the pathogenesis of, human ASD and SCZ. Overall, these data are the first to describe functional KCC2 genetic variants in human psychiatric disease, and suggest a compelling genetic overlap among distinct NDs. Furthermore, we show that CpG sites in the targeted-screened 3′ end of *SLC12A5*, as well as in the entire gene, are more commonly affected (i.e., disrupted or gained) in ASD cases compared to controls.

Genetic links among the different NDs have been previously shown. For example, chromosome 1q21.1 microdeletions exist in both ID and SCZ, and, interestingly, are often inherited from an unaffected or a mildly affected parent (Christiansen et al., 2004; Brunetti-Pierri et al., 2008; International-Schizophrenia-Consortium, 2008; Mefford et al., 2008; Stefansson et al., 2008). Duplications of this region are also associated with mild to moderate ID and ASD (Brunetti-Pierri et al., 2008; Mefford et al., 2008). Deletions of chromosome 16p13.11 have been associated with ID and ASD (Ullmann et al., 2007), and epilepsy (Heinzen et al., 2010; de Kovel et al., 2010), and duplications of this region have been documented in ID (Ullmann et al., 2007; Mefford et al., 2009), ASD (Ullmann et al., 2007) and SCZ (Kirov et al., 2009). In addition, mutations in *MECP2* [OMIM 300005] (Lam et al., 2000) and *SLC6A8* [OMIM 300036] (Salomons et al., 2001) have been found in both ID and ASD; and, mutations in *SHANK3* [OMIM 606230] have been identified in ASD (Durand et al., 2007), SCZ (Gauthier et al., 2010) and nonsyndromic ID cohorts (Hamdan et al., 2011). The impact of *de novo* SNVs (single nucleotide variants) on sporadic forms of common NDs have also been increasingly appreciated (Vissers et al., 2010; Girard et al., 2011; O’Roak et al., 2011, 2012; Xu et al., 2011; Iossifov et al., 2012; Neale et al., 2012; Sanders et al., 2012), and *de novo* ASD mutations have been identified in genes previously associated with other NDs, such as *FOXP1* [OMIM 605515], *GRIN2B* [OMIM 138252], *SCN1A* [OMIM 182389], and *LAMC3* [OMIM 604349] (O’Roak et al., 2011). No *de novo* variants were detected in this study.

Identifying rare variants that are associated with a complex trait is most currently performed through whole-exome sequencing since there is a potential to identify many genes that underlie the trait at a substantially lower cost compared to whole-genome sequencing; plus, exome variants offer a clear-cut functional annotation that can be predicted through many *in silico* bioinformatics programs with an accuracy of ~80% (Kiezun et al., 2012). However, with exome sequencing, a *p*-value of less than 2.5 × 10^−6^ is required to reach genome-wide significance; this value accounts for multiple testing by making a Bonferroni correction for 20,000 independent tests (1 test for each gene in the genome). Such a threshold is very conservative and unless a study is extremely large, significant *p*-values will not be reached. In fact, current exome sequencing studies of complex traits are under-powered since, at minimum, 10,000 individuals with a distinct phenotype are needed in order to achieve the necessary power (Kryukov et al., 2009; Kiezun et al., 2012). Furthermore, rare variants should be combined in a gene (or pathway) during association tests in order to reach sufficient power (Purcell et al., 2003; Kiezun et al., 2012).

A true understanding of how risk alleles interact and contribute to disease is yet to come. In a polygenic or oligogenic disease model, causality cannot be assigned to any one variant, but rather results from an individual’s variant pattern (Klassen et al., 2011). A recent exome sequencing paper aimed to demonstrate the polygenic burden of rare disruptive mutations in SCZ by noting the disruptive mutations that were distributed across many genes as well as enriched gene sets (Purcell et al., 2014). The ultimate goal is to identify all risk variants and to establish a computational modeling of biological networks in order to improve risk predictions based on a combination of alleles. Interestingly, Kiezun et al. (2012) noted that no individual gene-based test achieved significance after correction for multiple testing, again reiterating the need to increase sample size and/or take more targeted screening approaches to minimize multiple testing.

Here, we utilized a hypothesis-driven, targeted sequencing approach to search for variation in the KCC2 CTRD in different NDs given the known neurophysiological importance of this gene (Gagnon and Delpire, 2013), and the demonstrated critical role of our targeted region for the functional regulation of synapses (Chamma et al., 2012; Kahle et al., 2013). This approach previously enabled us to simplify the statistical analysis to identify the enrichment of NS alleles in the KCC2 CTRD in IGE cases vs. controls (*p-value* = 7.50 × 10^−3^; Kahle et al., 2014). Rare variation is enriched for evolutionarily deleterious variants, and we demonstrated that the KCC2 IGE variants (R952H and R1049C) impaired KCC2 function (Kahle et al., 2014). The initial targeted screening of the KCC2 CTRD in the FC cohort of ASD, SCZ, and ID did not generate any significant associations; however, it revealed that the functional variants overlap across different NDs, suggesting a role in disease pathogenesis, and the effects of these variants are likely dependent on the combination of other alleles within each individual. After combining our initial findings with the results of other exome sequencing projects (Iossifov et al., 2014), we were able to generate improved *p*-values due to the larger sample sets. It was after this analysis that a statistically significant excess of KCC2 CTRD variants in the targeted region was identified in ASD cases compared to controls, as well as an increase of variants that affected CpG sites. It should be noted that the authors recognize the disadvantages of comparing different exome sequencing data sets (SSC and EVS) that were analyzed using different pipelines; replication studies are warranted.

What is the functional impact of the discovered KCC2 variants? The KCC2 R952H and R1049C variants significantly decrease KCC2-mediated Cl^−^ extrusion capacity in neurons, render E_Gly_ less hyperpolarized compared to WT KCC2, decrease the level of KCC2 Ser940 phosphorylation, act in a dominant-negative manner, and decrease transporter plasmalemmal expression (R952H) or lower the intrinsic activity of transporters at the cell surface (R1049C). KCC2 R952H was also shown to substantially decrease dendritic spine density and alter spine morphology (Puskarjov et al., 2014). Given the evolutionary conservation of KCC2 R1048, the predicted pathogenicity of the R1048W substitution by multiple *in silico* algorithms, and the proximity of this variation to R1049, we anticipate the R1048W KCC2 variant detected in ASD, like R1049C, alters the intrinsic activity of KCC2 transporters at the cell surface. The clustering of the R1048 and R1049 variants suggests this region of KCC2 is particularly important for transporter regulation. Consistent with this is the proximity of the KCC2 “ISO” domain, encoded in amino acids 1022–1037, which is required for isotonic KCC2-mediated hyperpolarizing GABAergic transmission (Acton et al., 2012). These variants might change C-terminal protein structure and alter the function of the “ISO” domain, perhaps by disrupting the binding of key associated regulatory molecules.

From a pathophysiological standpoint, decreased KCC2-mediated Cl^−^ efflux in individuals carrying the KCC2 R952H, R1048W, and R1049C variants would be anticipated to increase intracellular [Cl^−^], raising the Cl^−^ reversal potential (E_Cl_) to less hyperpolarized potentials, and compromising GABA_A_R-mediated hyperpolarizing inhibition. In humans, KCC2 is developmentally upregulated, with low expression *in utero*, a rapid increase in expression around 40 postconceptional weeks, and progressively increasing levels of expression into adulthood (Dzhala et al., 2005). This pattern of expression drives the developmental switch of GABAergic signaling from depolarizing in early development to hyperpolarizing in adulthood, but the precise role of KCC2 or its disruption in neurodevelopment is unclear. The progressive developmental increase in KCC2 expression into adulthood might be expected to amplify the functional effects of KCC2 variants on CNS function over time. This may correlate with the age of onset of these neuropsychiatric disorders, as clinical symptomatology might manifest only after years of neurodevelopment over which deficits in KCC2 activity relative to normal become more pronounced. Further studies are indicated to explore this hypothesis.

The effects of KCC2 variants identified in this study might be similar, though less potent in magnitude given their heterozygousity, to phenotypes observed in mice with complete knockout or mild dysfunction of KCC2. Mice with complete KCC2 knockout die at birth from profound motor deficits that abolish respiratory function (Hekmat-Scafe et al., 2006, 2010; Tanis et al., 2009; Bellemer et al., 2011), but, interestingly, mice expressing hypomorphic alleles that reduce but do not abolish KCC2 activity demonstrate anxiety-like behavior, impaired spatial learning, and decreased seizure threshold (Woo et al., 2002; Tornberg et al., 2005; Zhu et al., 2008). Together, these data indicate that regulation of Cl^−^ homeostasis and GABAergic signaling by KCC2 plays a role in multiple functional systems of the CNS. These studies have also demonstrated that genetic KCC2 knockdown is not accompanied by compensatory changes in the expression of NKCC1 or other KCC isoforms, although post-translational compensatory regulation (e.g., phosphorylation) cannot be ruled out (Hubner et al., 2001; Woo et al., 2002; Tornberg et al., 2005).

Compellingly, CpG sites in the screened 3′ end of *SLC12A5*, as well as in the entire gene, are more commonly affected (i.e., disrupted or gained) in ASD cases compared to controls. Generally, DNA methylation occurs at CpG sites and plays a role in gene expression by suppressing gene transcription. Therefore, differences in methylation patterns between cases and controls could result in different gene expression patterns that increase risk to ASD. CpG islands, regions of CpG clusters that are associated with genes, are generally involved in transcription activation. Interestingly, there is a CpG island of ~2000bp at the 3′ end of *SLC12A5* that partially overlaps with our targeted-screened region. Furthermore, despite the fact that the variants detected in our targeted-screened region are outside that specific CpG island, most methylation differences between tissues and, perhaps even between patients and controls, actually occur at CpG sites at a short distance from the actual CpG island (Irizarry et al., 2009). It would be interesting to screen the entire *SLC12A5* locus (coding and non-coding regions) to determine if non-coding variants contribute towards a stronger association in ASD cases and controls. Additionally, carrying out the same analysis in an SCZ case/control cohort would be valuable.

Lastly, our results are interesting given the recent data from the Fragile X and valproate mouse models of autism, which demonstrate an abnormally prolonged elevation of [Cl^−^]_i_ in developing CNS neurons that delays the normal ontogenic switch to GABA inhibition (He et al., 2014). Maternal oxytocin during delivery normally mediates the hyperpolarization of E_GABA_, but this effect is abolished in the these models (Tyzio et al., 2014). Our data here suggest a compelling hypothesis that abnormally delayed KCC2-mediated Cl^−^ extrusion during the developmental GABA excitatory-inhibitory sequence might be a genetically programmed factor contributing to ASD. Further investigation into the roles of KCC2 in ASD and SCZ, and potentially other NDs, may offer new therapeutic strategies.

## Conflict of Interest Statement

The authors declare that the research was conducted in the absence of any commercial or financial relationships that could be construed as a potential conflict of interest.

## Footnotes

^1^http://evs.gs.washington.edu/EVS/
